# Systemic immune-inflammation index is associated with aneurysmal wall enhancement in unruptured intracranial fusiform aneurysms

**DOI:** 10.3389/fimmu.2023.1106459

**Published:** 2023-01-27

**Authors:** Fei Peng, Jiaxiang Xia, Hao Niu, Xin Feng, Tianheng Zheng, Xiaoxin He, Boya Xu, Xuge Chen, Peng Xu, Hong Zhang, Jigang Chen, Xin Tong, Xiaoyan Bai, Zhiye Li, Yonghong Duan, Binbin Sui, Xingquan Zhao, Aihua Liu

**Affiliations:** ^1^ Beijing Neurosurgical Institute and Beijing Tiantan Hospital, Capital Medical University, Beijing, China; ^2^ Neurosurgery Center, Department of Cerebrovascular Surgery, Engineering Technology Research Center of Education Ministry of China on Diagnosis and Treatment of Cerebrovascular Disease, Zhujiang Hospital, Southern Medical University, Guangzhou, Guangdong, China; ^3^ College of Integrated Chinese and Western Medicine, Jining Medical University, Jining, China; ^4^ Department of Neurosurgery, The Second Affiliated Hospital, Hengyang Medical School, University of South China, Hengyang, Hunan, China; ^5^ Operating Room, Heze Municipal Hospital, Heze, Shandong, China; ^6^ Tiantan Neuroimaging Center of Excellence, China National Clinical Research Center for Neurological Diseases, Beijing, China; ^7^ Department of Neurology, Beijing Tiantan Hospital, Capital Medical University, Beijing, China

**Keywords:** intracranial aneurysm, inflammation, aneurysmal wall enhancement, magnetic resonance imaging, immune

## Abstract

**Introduction:**

Inflammation plays a key role in the progression of intracranial aneurysms. Aneurysmal wall enhancement (AWE) correlates well with inflammatory processes in the aneurysmal wall. Understanding the potential associations between blood inflammatory indices and AWE may aid in the further understanding of intracranial aneurysm pathophysiology.

**Methods:**

We retrospectively reviewed 122 patients with intracranial fusiform aneurysms (IFAs) who underwent both high-resolution magnetic resonance imaging and blood laboratory tests. AWE was defined as a contrast ratio of the signal intensity of the aneurysmal wall to that of the pituitary stalk ≥ 0.90. The systemic immune-inflammation (SII) index (neutrophils × platelets/lymphocytes) was calculated from laboratory data and dichotomized based on whether or not the IFA had AWE. Aneurysmal symptoms were defined as sentinel headache or oculomotor nerve palsy. Multivariable logistic regression and receiver operating characteristic curve analyses were performed to determine how well the SII index was able to predict AWE and aneurysmal symptoms. Spearman’s correlation coefficients were used to explore the potential associations between variables.

**Results:**

This study included 95 patients, of whom 24 (25.3%) presented with AWE. After adjusting for baseline differences in neutrophil to lymphocyte ratios, leukocytes, and neutrophils in the multivariable logistic regression analysis, smoking history (P = 0.002), aneurysmal symptoms (P = 0.047), maximum diameter (P = 0.048), and SII index (P = 0.022) all predicted AWE. The SII index (P = 0.038) was the only independent predictor of aneurysmal symptoms. The receiver operating characteristic curve analysis revealed that the SII index was able to accurately distinguish IFAs with AWE (area under the curve = 0.746) and aneurysmal symptoms (area under the curve = 0.739).

**Discussion:**

An early elevation in the SII index can independently predict AWE in IFAs and is a potential new biomarker for predicting IFA instability.

## Introduction

Intracranial aneurysm (IA) rupture results in aneurysmal subarachnoid hemorrhage, a severe event with high rates of mortality and disability (1). Several factors, including hemodynamics, biomechanical remodeling, and inflammation, contribute to IA instability; inflammatory processes in the aneurysmal wall play a particularly critical role (2–4). In a pathological study, it was reported that the status of inflammatory processes in the aneurysmal wall correlates well with aneurysmal wall enhancement (AWE) in high-resolution magnetic resonance imaging (HR-MRI) (5). Inflammatory processes in the aneurysmal wall also involve the local activation of the immune system, including neutrophil influx followed by the infiltration of macrophages and other inflammatory cells (6, 7). It has been reported that cytokine interleukin-10 and the neutrophil to lymphocyte ratio (NLR) are associated with AWE in HR-MRI (8, 9). Given that AWE has emerged as an imaging biomarker of IA instability, a blood inflammatory index may reflect inflammatory processes in the aneurysmal wall and might thus serve as a new biomarker of IA instability. However, the particular comprehensive blood inflammatory index that might predict AWE remains to be investigated.

The systemic immune-inflammation (SII) index indicates the systemic inflammatory response and can be used to predict outcomes in patients with aneurysmal subarachnoid hemorrhage (10). Unlike the NLR, the SII index incorporates platelet counts and outperforms the NLR for predicting poor outcomes of aneurysmal subarachnoid hemorrhage, especially for IAs with a strong interaction between inflammation and thrombosis (10). Intracranial fusiform aneurysms (IFAs) are a rare type of IA that accounts for just 3%–13% of all IAs (11). It has been reported that IFAs have a high incidence of mural thrombus and diffuse arterial enhancement, which suggests extensive inflammation of the entire involved artery (12, 13). We therefore hypothesized that an elevated SII index might be better at predicting AWE in IFA than the NLR and other blood inflammatory indexes.

In the current study, we thus aimed to investigate the association between the SII index and AWE in IFAs. Additionally, to further investigate the potential role of the SII index in predicting IA instability, we explored the association between the SII index and aneurysmal symptoms.

## Materials and methods

### Study population and data collection

One hundred twenty-two patients with IFAs who underwent HR-MRI and blood laboratory tests at admission were retrospectively recruited from August 2016 to April 2022 in Beijing Tiantan Hospital. The baseline characteristics of patients, including age, sex, hypertension, diabetes, dyslipidemia, and smoking history, were recorded from medical records or obtained *via* telephone follow-up. Symptomatic IAs were defined as patients who had sentinel headache or oculomotor nerve palsy, which reportedly strongly indicate unstable IAs (14). IFAs were classified into three subtypes as in previous studies (13): fusiform type, dolichoectatic type, and transitional type. The study exclusion criteria were: 1) patients aged under 18 years; 2) patients with a history of interventional or surgical treatment; 3) patients with a history of acute or chronic infectious inflammation (e.g., pneumonia or urinary tract infection), autoimmune disease, heart disease, hematological disease, or cancer; 4) patients in whom IFAs coexisted with other intracranial vascular diseases (e.g., dissecting aneurysms, moyamoya disease, arteriovenous malformations, or dural arteriovenous fistulas); 5) patients from whom venous blood samples were not obtained on admission; and 6) patients with incomplete medical records or laboratory indicators or with poor image quality for MRI.

### Blood laboratory test

The blood laboratory test within 24 h after admission included white blood cell, lymphocyte, neutrophil, and platelet counts. NLR was calculated as the ratio of neutrophils to lymphocytes (9) and the SII index was calculated as ([platelet count × absolute neutrophil count/absolute lymphocyte count]/1000) (10).

### HR-MRI protocol

All MRI scans were performed using a 3.0 T MR scanner (Ingenia CX, Philips Healthcare; Trio-Tim, Siemens Healthcare; or Discovery 750, GE Healthcare) equipped with a 32-channel head coil. Three-dimensional (3D) time-of-flight magnetic resonance angiography and 3D-T1-weighted imaging (WI) sequences were combined to confirm IFAs. The pre-contrast 3D-T1WI (VISTA/SPACE/CUBE) and post-contrast 3D-T1WI (VISTA/SPACE/CUBE) sequences were included in the HR-MRI protocol with a voxel size of 0.7 × 0.7 × 0.7 mm^3^. After scanning the pre-contrast T1W images, each patient was administered a Gd injection (0.1 mmol/kg gadopentetate dimeglumine, Magnevist; Bayer Schering Pharma AG). Six minutes later, post-contrast 3D-T1WI images were obtained.

### AWE Measurement

AWE quantification was performed using Horos (https://horosproject.org/). The spatial position of each IFA was determined using 3D multiplanar reconstruction. The cross-section of each IFA was identified by the transverse plane at the site of maximal dilation in 3D space, while the longitudinal section was identified by the long-axis plane, which incorporated the adjacent arteries with 1.5× the normal artery as the boundary. The maximum diameter (D_max_) of an IFA, measured on pre-contrast T1WI, was defined as the maximal diameter of the cross-section (15).

**Figure 1:**
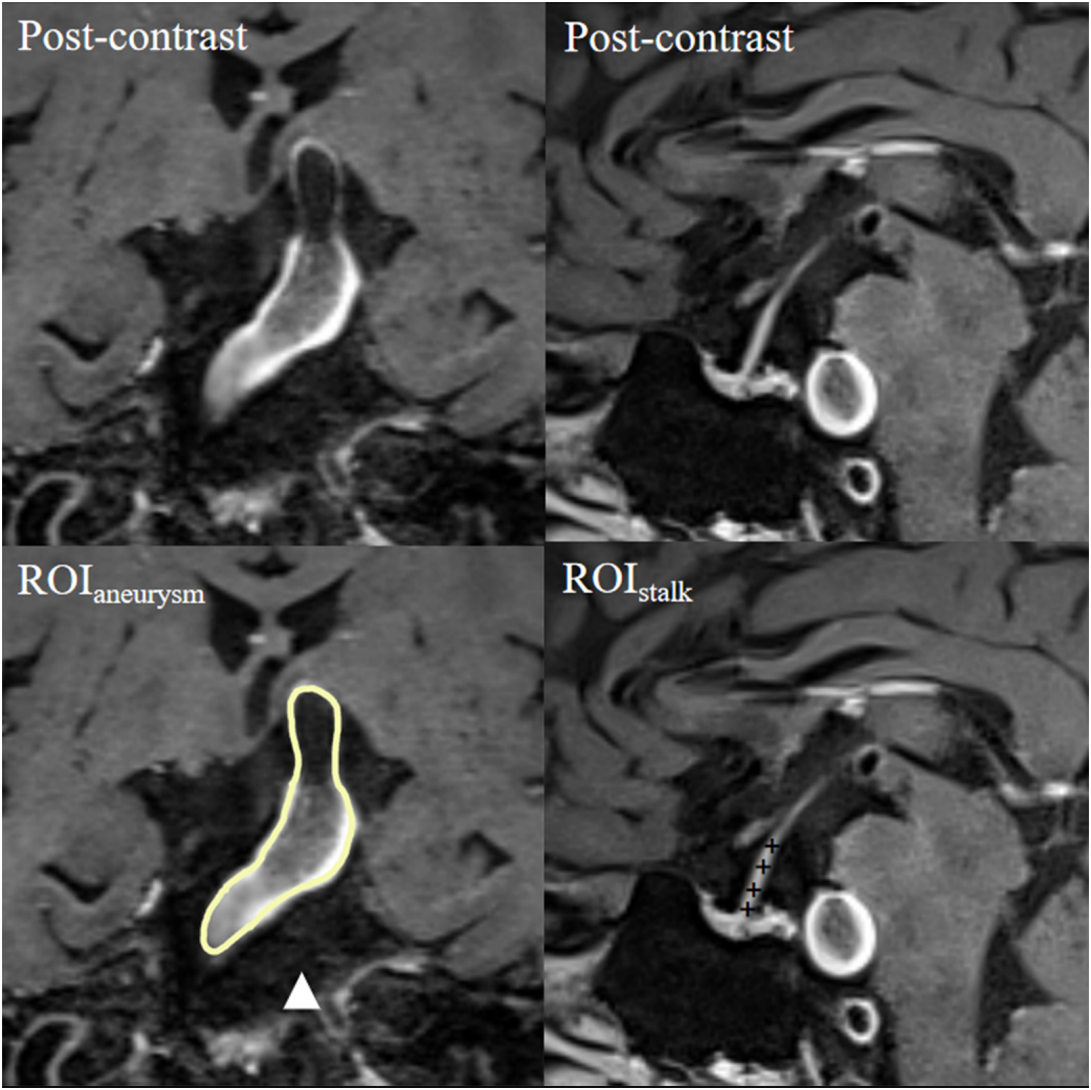
Representative case of aneurysmal wall enhancement assessment. The first row shows post-contrast T1-weighted images of a basilar fusiform aneurysm and a sagittal view of the pituitary stalk. The second row shows the ROIs of the aneurysm and pituitary stalk. The average signal intensity of the ROI of the aneurysm (ROI_aneurysm_) and the average signal intensity of four randomized points inside the ROI of the pituitary stalk (ROI_stalk_) were measured. CR_stalk_ was calculated as the contrast ratio of the signal intensity of ROI_aneurysm_ to ROI_stalk_. CR, contrast ratio; ROI, region of interest.

To determine and delineate a clear aneurysmal wall, we used the co-registration of 3D time-of-flight magnetic resonance angiography and pre- and post-contrast 3D-T1WI (VISTA/SPACE/CUBE); to eliminate pseudo-enhancement, we used a method described in our previous studies (16). The regions of interest—the aneurysmal wall and pituitary stalk—were located in their respective longitudinal sections (17). Each region of interest was first determined by one experienced neuroradiologist (with 20 years of experience in neuroradiology) and then by another two experienced radiologists (with 20 and 15 years of experience in neuroradiology, respectively). The radiologists were blinded to patient characteristics and performed the measurements independently. The aneurysm-wall-to-pituitary-stalk contrast ratio (CR_stalk_), calculated as the ratio of the signal intensity of the aneurysmal wall to the average signal intensity of four randomized points of the pituitary stalk, was used to quantify AWE (**Figure 1**). AWE was defined as a CR_stalk_ ≥ 0.90, as reported in our previous studies (17). Any discrepancies in AWE as evaluated by the second two neurologists were resolved by the first neurologist; when the AWE evaluations of the second two neurologists were consistent, the CR_stalk_ value of the neurologist with more years of experience in neuroradiology was applied in the analysis.

### Statistical analysis

SPSS version 23.0 (IBM) was used to conduct all statistical analyses. Continuous variables are presented as the mean ± standard deviation (SD). The Mann–Whitney test or Kruskal–Wallis H test was used to compare continuous variables. Categorical variables were compared using the chi-squared test or Fisher’s exact test. To investigate the independent predictors of AWE or symptomatic IFAs, the baseline patient characteristics, IFAs, and laboratory indicators were first subjected to univariate analysis. Next, variables with P < 0.05 were added to the logistic regression analysis. Spearman’s correlation coefficient (rs) was used to explore the associations between these variables. Intraclass correlation coefficient, using a two-way random effects model, absolute agreement, and single rater/measurement (18), was used to assess the interobserver reliability of CR_stalk_ measurements by the two radiologists. Receiver operating characteristic curve analysis was performed to determine how well the SII index was able to predict AWE or symptomatic IFAs. The maximum Youden’s index was used to identify the cut-off value. A value of P < 0.05 indicates significance.

## Results

**Figure 2:**
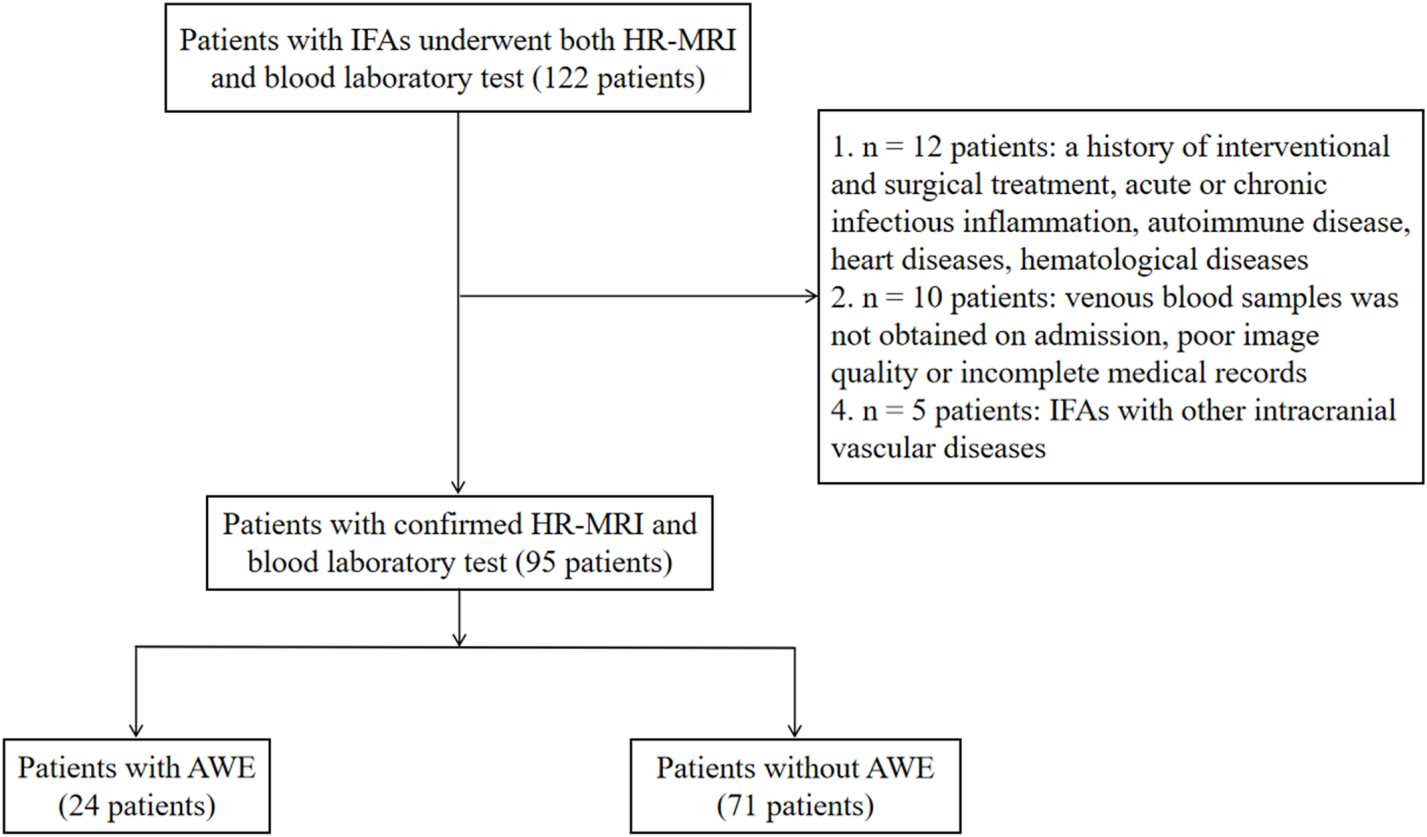
Patient inflow chart. AWE, aneurysmal wall enhancement; HR-MRI, high-resolution magnetic resonance imaging; IFAs, intracranial fusiform aneurysms.

In the database, we identified 122 patients who underwent both HR-MRI and blood laboratory tests. Of these, 95 (77.9%) met the inclusion criteria (**Figure 2**). Twenty-seven patients were excluded: 12 with acute or chronic infectious inflammation, autoimmune disease, heart disease, hematological disease, or a history of interventional and surgical treatment; 10 without venous blood samples obtained on admission or with poor image quality or incomplete medical records; and 5 with IFAs that coexisted with other intracranial vascular diseases.

The mean age was 52.04 ± 11.11 years, and 66 patients (69.5%) were male. Thirty-one patients (32.6%) presented with aneurysmal symptoms and 64 (67.4%) did not. The mean aneurysm size was 9.60 ± 4.23 mm with a mean CR_stalk_ of 0.71 ± 0.25. Of the IFAs, 24 (25.3%) had AWE (CR_stalk_ ≥ 0.90) and 71 (74.7%) had no AWE (CR_stalk_ < 0.90). Seventy-nine IFAs (83.2%) were located in the posterior circulation. The baseline characteristics of patients and IFAs are shown in **Table 1**.

**Table 1:** Baseline characteristics of IFA patients with and without AWE.

Variable	All, N = 95	AWE, N = 24	No AWE, N = 71	P value
Demographic data				
Age, years	52.04 ± 11.11	49.92 ± 9.69	52.76 ± 11.5	0.125
Male patients, n (%)	66 (69.5)	20 (83.3%)	46 (64.8%)	0.124
Risk factors				
Hypertension	50 (52.6)	14 (58.3%)	36 (50.7%)	0.638
Diabetes	6 (6.3)	1 (4.2%)	5 (7.0%)	1.000
Dislipidemia	25 (26.3)	4 (16.7%)	21 (29.6%)	0.287
Smoking history	43 (45.3)	18 (75.0%)	25 (35.2%)	0.001*
Symptoms	31 (32.6)	14 (58.3%)	17 (23.9%)	0.005*
Aneurysm location				0.063
Anterior circulation	16 (16.8)	1 (4.2%)	15 (21.1%)	
Posterior circulation	79 (83.2)	23 (95.8%)	56 (78.9%)	
Types of IFAs				0.063
Fusiform	69 (72.6)	13 (54.2%)	56 (78.9%)	
Dolichoectatic	90 (9.5)	4 (16.7%)	6 (7.0%)	
Transitional	17 (17.9)	7 (29.2%)	10 (14.1%)	
D_max_, mean ± SD, mm	9.60 ± 4.23	11.76 ± 4.23	8.87 ± 4.0	0.001*
Mural thrombus, N (%)	29 (30.5)	11 (45.8%)	18 (25.4%)	0.075
Atherosclerosis, N (%)	43 (45.3)	15 (62.5%)	28 (39.4%)	0.060
Blood examinations, mean ± SD				
Leukocytes, 10^9^/L	6.57 ± 1.90	7.61 ± 2.14	6.21 ± 1.69	0.009*
Neutrophils, 10^9^/L	4.10 ± 1.54	5.07 ± 1.92	3.78 ± 1.24	0.004*
Lymphocytes, 10^9^/L	1.91 ± 0.66	1.87 ± 0.67	1.92 ± 0.65	0.884
NLR	2.42 ± 1.45	3.28 ± 2.26	2.13 ± 0.89	0.014*
Platelets, 10^9^/L	0.23 ± 0.05	0.24 ± 0.04	0.22 ± 0.06	0.053
SII index, 10^9^/L	0.53 ± 0.31	0.76 ± 0.45	0.45 ± 0.18	< 0.001*

*Significant values. IFAs, intracranial fusiform aneurysms; AWE, aneurysmal wall enhancement; D_max_, maximum diameter; SD, standard deviation; NLR, neutrophil to lymphocyte ratio; SII, systemic immune-inflammation index.

**Figure 3:**
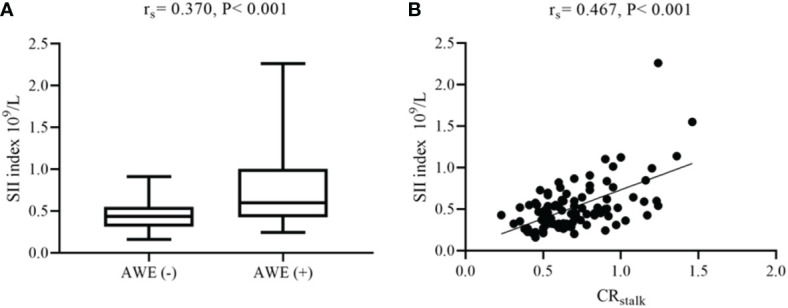
Associations between AWE, CR_stalk_, and the SII index. **(A)** Box plot comparing the SII index for AWE and non-AWE aneurysms. **(B)** Scatter plot of the SII index and CR_stalk_. AWE, aneurysmal wall enhancement; CR_stalk_, contrast ratio of the aneurysmal wall to the pituitary stalk; SII, systemic immune-inflammation index.

**Figure 4:**
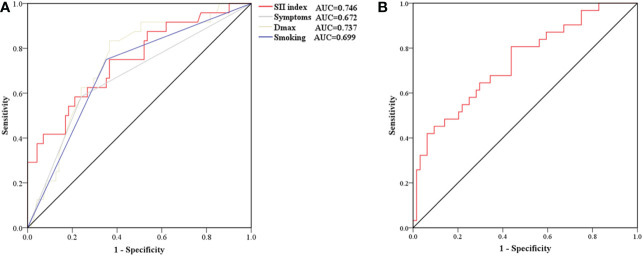
Receiver operating characteristic curve for determining the presence of aneurysmal wall enhancement **(A)** and aneurysmal symptoms **(B)**. **(A)** The AUC of the SII index, smoking, and D_max_ were 0.746 (sensitivity 75.0%, specificity 63.4%), 0.743 (sensitivity 75.0%, specificity 64.8%), and 0.699 (sensitivity 83.3%, specificity 63.4%), respectively. **(B)** The AUC of the SII index was 0.739 (sensitivity 80.6%, specificity 56.2%). AUC, area under the curve; D_max_, maximum diameter; SII, systemic immune-inflammation.

The different characteristics of patients with and without AWE were then investigated. Compared with those without AWE, patients with AWE tended to have a history of smoking (75.0% vs 35.2%, P = 0.001), aneurysmal symptoms (58.3% vs 23.9%, P = 0.001), and a higher D_max_ (11.76 vs 8.87, P < 0.001). We then investigated differences in blood examinations on admission. Compared with those without AWE, patients with AWE had higher median total leukocytes (7.61 vs 6.21 × 10^9^/L, P = 0.009), absolute neutrophil count (5.07 vs 3.78 × 10^9^/L, P = 0.004), NLR (3.28 vs 2.13, P = 0.014), and SII index (0.76 vs 0.45 × 10^9^/L, P < 0.001). After adjusting for baseline differences in NLR, leukocytes, and neutrophils in the multivariable logistic regression analysis, smoking history (P = 0.002), D_max_ (P = 0.048), aneurysmal symptoms (P = 0.047), and SII index (P = 0.022) all predicted AWE **(** **Table 2** **)**. Next, box plots and scatter plots were used to analyze the associations between the SII index and AWE and the SII index and CR_stalk_, respectively **(** **Figure 3** **)**. Receiver operating characteristic curve analysis was then used to identify how well different variables were able to distinguish between IFAs with or without AWE **(** **Figure 4A** **)**. The SII index was able to differentiate between IFAs with and without AWE (area under the curve = 0.746) with an optimal cutoff value of 0.49 × 10^9^/L.

**Table 2:** Multivariable analysis for predictors associated with AWE.

Variables	Beta	OR (95% CI)	P value
History of smoking	-2.293	0.101 (0.024-0.432)	0.002*
Symptoms	-1.445	0.236 (0.057-0.979)	0.047*
D_max_, mm	0.165	1.179 (1.001-1.388)	0.048*
Leukocytes (10^9^/L), perSD	2.171	8.769 (0.730-105.292)	0.087
Neutrophils (10^9^/L), perSD	-2.807	0.060 (0.002-1.711)	0.100
NLR, per SD	0.934	2.545 (0.342-18.968)	0.362
SII index (10^9^/L), perSD	2.302	9.997 (1.385-72.169)	0.022*

AWE, aneurysmal wall enhancement; OR, odds ratio; CI, confidence interval; D_max_, maximum diameter; SD, standard derivation; NLR, neutrophil to lymphocyte ratio; SII, systemic immune-inflammation index; *P < 0.05.

Risk factors of aneurysmal symptoms were also investigated. In the univariate analysis, age (P = 0.009), CR_stalk_ (P < 0.001), AWE (P = 0.005), neutrophils (P = 0.005), NLR (P < 0.001), and the SII index (P < 0.001) were significantly associated with aneurysmal symptoms **(** **Table 3** **)**. In the multivariate analysis, only the SII index (P = 0.038) was an independent predictor of aneurysmal symptoms **(** **Table 4** **)**. To determine how well the SII index was able to distinguish between symptomatic and asymptomatic IFAs, receiver operating characteristic curve analysis was performed **(** **Figure 4B** **)**. The SII index was able to differentiate between patients with and without aneurysmal symptoms (area under the curve = 0.739) with an optimal cutoff value of 0.44 × 10^9^/L.

**Table 3:** Baseline characteristics of IFA patients with and without aneurysmal symptoms.

Variable	All, N = 95	Symptomatic, N = 31	Asymptomatic, N = 64	P value
Demographic data				
Age, years	52.04 ± 11.11	48.68 ± 10.38	53.7 ± 11.16	0.009*
Male patients, n (%)	66 (69.5)	19 (61.3%)	47 (73.4%)	0.244
Risk factors				
Hypertension	50 (52.6)	15 (48.4%)	35 (54.7%)	0.663
Diabetes	6 (6.3)	1 (3.2%)	5 (7.8%)	0.660
Dislipidemia	25 (26.3)	6 (19.4%)	19 (29.7%)	0.330
Smoking history	43 (45.3)	14 (45.2%)	29 (45.3%)	1.000
Aneurysm location				0.771
Anterior circulation	16 (16.8)	6 (19.4%)	10 (15.6%)	
Posterior circulation	79 (83.2)	25 (80.6%)	54 (84.4%)	
Types of IFAs				0.171
Fusiform	69 (72.6)	19 (61.3%)	50 (78.1%)	
Dolichoectatic	90 (9.5)	5 (16.1%)	4 (6.3%)	
Transitional	17 (17.9)	7 (22.6%)	10 (15.6%)	
CR_stalk_, mean ± SD	0.71 ± 0.25	0.83 ± 0.25	0.65 ± 0.23	< 0.001*
AWE, N (%)	24 (25.3)	14 (45.2)	10 (15.6)	0.005*
D_max_, mean ± SD, mm	9.60 ± 4.23	10.29 ± 4.58	9.27 ± 4.05	0.340
Mural thrombus, N (%)	29 (30.5)	12 (38.7%)	17 (26.6%)	0.244
Atherosclerosis, N (%)	43 (45.3)	18 (58.1%)	25 (39.1%)	0.123
Blood examinations, mean ± SD				
Leukocytes, 10^9^/L	6.57 ± 1.90	7.13 ± 2.24	6.29 ± 1.67	0.067
Neutrophils, 10^9^/L	4.10 ± 1.54	4.82 ± 1.89	3.76 ± 1.21	0.005*
Lymphocytes, 10^9^/L	1.91 ± 0.66	1.73 ± 0.51	1.99 ± 0.70	0.060
NLR	2.42 ± 1.45	3.01 ± 1.67	2.13 ± 1.24	< 0.001*
Platelets, 10^9^/L	0.23 ± 0.05	0.23 ± 0.05	0.22 ± 0.05	0.487
SII index, 10^9^/L	0.53 ± 0.31	0.70 ± 0.39	0.45 ± 0.21	< 0.001*

IFAs, intracranial fusiform aneurysms; CR_stalk_, contrast ratio of the aneurysmal wall to the pituitary stalk; SD, standard deviation; AWE, aneurysmal wall enhancement; D_max_, maximum diameter; NLR, neutrophil to lymphocyte ratio; SII, systemic immune-inflammation index; *P < 0.05.

**Table 4:** Multivariable analysis for predictors associated with aneurysmal symptoms.

Variables	Beta	OR (95% CI)	P value
CR_stalk_	1.995	7.354 (0.736-73.444)	0.089
Age, year	-0.037	0.964 (0.923-1.006)	0.091
Neutrophils (10^9^/L), perSD	0.028	1.028 (0.476-2.220)	0.944
NLR, per SD	-0.246	0.782 (0.261-2.342)	0.661
SII index (10^9^/L), perSD	0.778	2.176 (1.046-4.528)	0.038*

OR, odds ratio; CI, confidence interval; CR_stalk_, contrast ratio of the aneurysmal wall to the pituitary stalk; SD, standard derivation; NLR, neutrophil to lymphocyte ratio; SII, systemic immune-inflammation index; *P < 0.05.

### Reproducibility of measurements

For the CR_stalk_ measurements from the 95 included patients, interobserver agreement was excellent (intraclass correlation coefficient = 0.87 [95% confidence interval, 0.74–0.93]).

## Discussion

It is increasingly recognized that intracranial aneurysm is a disease that is driven by chronic inflammation (2, 4, 7). The SII index is a peripheral blood inflammatory index that is used to evaluate systemic inflammation (10), whereas AWE is regarded as a biomarker of aneurysmal wall inflammation (5). In the present study, we first demonstrated that the SII index was elevated in IFAs with AWE compared with those without AWE. We then revealed that the SII index was an independent predictor of both AWE and aneurysmal symptoms.

Although serum predictors such as cytokine interleukin-10 and the NLR have been reported to predict AWE (8, 9), a comprehensive blood inflammatory index that predicts AWE remains lacking; this is likely because inflammatory processes in the aneurysmal wall are complex (2). The SII index, which integrates information about neutrophils, lymphocytes, and platelets, may add accuracy for predicting AWE (10).

Previous studies have demonstrated that because systemic inflammation is frequent after aneurysmal subarachnoid hemorrhage (aSAH), the SII index, which reflects the systemic inflammation burden, can be used to independently predict the occurrence of vasospasm after aSAH (10). While the present study concentrated on unruptured IFAs, the positive association between the SII index and both AWE and CR_stalk_ may suggest a potential role for the blood inflammatory microenvironment in driving inflammatory processes in the aneurysmal wall. In the study of human IA specimens, inflammatory cells—including macrophages, neutrophils, and lymphocytes—were found in the aneurysmal wall (6). Neutrophils are considered an important source of matrix metalloproteinases and elastase, which are vital for aneurysm formation (19, 20). As is reported that they played a key role in the formation and progression of abdominal artery aneurysms in an animal model (21). In addition, lymphocytes can produce proinflammatory cytokines and may be associated with aneurysm rupture (22). NLR, which integrates changes in both neutrophils and lymphocytes, was reportedly to be associated with circumferential AWE (9). In the present study, the NLR was significantly associated with AWE, whereas the SII index (which contains additional information about platelets compared with the NLR) was an independent predictor of AWE. Apart from hemostatic properties, platelets can release inflammatory mediators, which may change the response of leukocyte and endothelial cells to inflammatory stimulation (23). In addition, platelets can also directly affect adaptive immune responses (24). Therefore, platelets coordinate both the inflammation and immune responses, and it is reported that platelets expedite vascular inflammation (25). Notably, IFAs have been reported to have large AWE surface areas, which suggests that extensive inflammatory processes may occur in the aneurysmal wall (17, 26). Additionally, it has been reported that nearly 50% of all IFAs have mural thrombus (13, 17), while platelet aggregation promotes the formation of mural thrombus (27, 28). Therefore, as an integrated index (combining neutrophils, lymphocytes, and platelets), the SII index tended to better predict AWE in IFAs than other indexes (including NLR and other individual components of the SII index) in the current study.

Three other independent predictors of AWE included D_max_, aneurysmal symptoms, and smoking history. D_max_ is a morphological parameter of aneurysm size in the cross-section, which may increase with the growth and progression of an IFA in the cross-sectional plane (29). Aneurysm size was reported to be positively associated with AWE (12, 30), while it is reported that AWE is the predictor of aneurysm growth (31). Therefore, D_max_ and AWE may also be positively correlated. Fu et al. demonstrated that aneurysmal symptoms are independently associated with AWE in saccular aneurysms (14). Cao et al. reported that AWE in non-saccular vertebrobasilar aneurysms was relevant to clinical symptoms (15). Our previous studies also indicated that both D_max_ and aneurysmal symptoms independently predicted AWE in IFAs (17, 29). Although many previous studies have investigated the predictors of AWE (3, 12, 14, 15, 17), no study has investigated the association between smoking history and AWE in IFAs. Smoking reportedly promotes endothelial dysfunction and atherosclerosis formation in the aneurysmal wall; these pathological changes have been reported to correlate well with AWE (5, 32). Another reason may be that patients with IFAs are mostly male (69.5% in this study) (13), who are more likely to smoke than females.

In the present study, levels of peripheral blood inflammatory indexes (including neutrophils, the NLR, and the SII index) were higher in patients with symptomatic IFAs than in those with asymptomatic IFAs. These findings indicate that systemic inflammation may promote AWE and IFA progression. Because the SII index was also identified as an independent predictor of aneurysmal symptoms, the SII index may therefore be a new biomarker of unstable IFAs; however, this requires further validation in a larger cohort. Based on the complex course of inflammatory processes that involve inflammatory cells, cytokines, and other agents in the aneurysmal wall, a more comprehensive and synthesized blood inflammatory index is needed.

This study has several clinical implications. First, the SII index may be a new biomarker of symptomatic IFAs for patient screening. Second, because the cutoff values of the SII index for predicting AWE and aneurysmal symptoms were 0.49 and 0.45 × 10^9^/L, respectively, an unruptured IFA with an SII index above these values may indicate its instability, especially in patients without clear symptoms or AWE. Third, the SII index may need to be closely monitored and controlled at a relatively lower level in patients with IFAs in future studies. Finally, patients with IFAs are advised to quit smoking, because smoking may contribute to AWE, which acts as a new imaging biomarker of aneurysm instability.

The major advantage of the present investigation is that it is the first study to investigate the association between the SII index and AWE in IFA using non-invasive tools. However, several limitations exist. First, this study involved a single center only and had a retrospective design and limited sample size. Second, three types of MRI scans with a relatively large voxel size were used; this may lead to bias. Third, this study lacks histological verification. Fourth, studies on 3D view of the entire aneurysm wall enhancement need to be carried out in future studies. Finally, other blood inflammatory factors such as atherosclerotic proteins and cytokines should be combined in future studies.

## Conclusions

In summary, we identified correlations between blood inflammatory variables in peripheral blood and AWE in IFAs. The SII index was associated with AWE and aneurysmal symptoms of IFAs, and is thus a potential new biomarker of IFA instability, although this needs further validation in future studies.

## Data availability statement

The original contributions presented in the study are included in the article/supplementary material. Further inquiries can be directed to the corresponding authors.

## Ethics statement

The studies involving human participants were reviewed and approved by Ethics Committee of Beijing Tiantan Hospital. The patients/participants provided their written informed consent to participate in this study.

## Author contributions

FP and JX: conception and design. HN, XF, TZ, XH, BX, PX, HZ, JC, and XT: acquisition of data. YD, and BS: analysis and interpretation of data. FP: drafting the article. XC, XB, and ZL: technical supports. XZ and AL: study supervision. All authors contributed to the article and approved the submitted version.
